# Application of a risk score model based on tyrosine-related genes in the prognosis and treatment of patients with lung adenocarcinoma

**DOI:** 10.3389/fimmu.2025.1667473

**Published:** 2025-11-04

**Authors:** Huan Wang, Yuebei Li, Ke Liu, Xinyuan Fan

**Affiliations:** ^1^ Department of Respiratory & Critical Care Medicine, The First Affiliated Hospital of Nanjing Medical University, Nanjing, China; ^2^ Department of Oncology, Xiaoxian People’s Hospital, Suzhou, China; ^3^ Department of Respiratory & Critical Care Medicine, Xuzhou Central Hospital, Xuzhou, China

**Keywords:** LUAD, tyrosine, immune, MYO6, signature

## Abstract

**Background:**

Tyrosine is associated with alterations in the tricarboxylic acid cycle in lung cancer, and exploring tyrosine-related genes (TRGs) has the potential to contribute to the construction of new sensitive prognostic biomarkers for patients with lung adenocarcinoma (LUAD).

**Method:**

Lung cancer prognosis model was constructed by Lasso Cox regression, univariate and multivariate COX regression, GSEA, TIDE. Potential drugs were screened and drug sensitivities analyzed by the pRRophetic software package. And the role of MYO6 in lung cancer was confirmed by experiments *in vitro*.

**Results:**

We identified 7 TRG risk score models (ZFP3, MEAK7, MMUR1, GTF3C6, MYO6, MAPK1IP1L and VAX1) for the diagnosis and prognosis of patients with LUAD. ROC curves and the C-index suggested that the risk score had more reliable diagnostic significance and could more accurately predict the prognosis of LUAD patients. The nomogram model was constructed with risk scores, which can be used to predict an individual and visualize the correlation between the total score and the predicted outcome more intuitively. Additionally, it has an impact on immunotherapy efficacy, tumor mutation burden and drug sensitivity. In addition, high expression of myosin VI (MYO6) was related to tumor proliferation and metastasis *in vitro*.

**Conclusion:**

In conclusion, the risk scores constructed from seven TRGs have great potential for survival prognosis, immunotherapy response and drug sensitivity. MYO6 plays an oncogenic role in promoting proliferation and metastasis in patients with LUAD, which provides a new theoretical basis for the diagnosis and treatment of LUAD patients.

## 1 Introduction

Lung cancer is one of the leading causes of cancer-associated mortality worldwide (1). The proportion of non-small cell lung cancer (NSCLC) cases is approximately 85%, and lung adenocarcinoma (LUAD) accounts for the majority of NSCLC cases, with a low five-year survival rate (2). Although much progress has been made in lung cancer treatment in recent years, problems such as drug resistance due to tumor heterogeneity still present many challenges for lung cancer treatment (3). Therefore, there is an urgent need for a comprehensive study at the gene level to develop biomarkers for predicting prognosis and effective tools for evaluating lung cancer treatment.

Tumor growth relies on oncogene-driven reprogramming of cellular metabolism, which enables cancer cells to absorb nutrients and promote proliferation (4). With the exploration of metabolic processes such as the Warburg effect, the tumor tricarboxylic acid cycle, and the pentose phosphate pathway, the relationships between the metabolic characteristics of tumors and tumor development have become clearer (5). Amino acids, as one of the main components of cellular metabolism, are important targets of antitumor drugs and strongly influence cell growth, development and proliferation (6). An increasing number of studies have shown that targeting amino acid metabolism has great potential for improving cancer therapy (7, 8). The latest study indicates that amino acid metabolism-related genes can serve as prognostic indicators for LUAD (9). Additionally, research shows that the insulin receptor, acting as a tyrosine protein kinase, can translocate to the nucleus of LUAD cells to promote their proliferation (10).

Tyrosine, an aromatic nonessential amino acid, acts as a building block for important chemicals that play crucial roles in metabolism in humans and is commonly used as a nutritional supplement for patients with phenylketonuria (11, 12). Studies have shown that tyrosine is associated with alterations in the tricarboxylic acid cycle in cancer (13, 14) and that reduced tyrosine levels may be due to metabolic disturbances caused by colorectal tumor progression (15). In addition, tyrosine may participate in the proliferation of tumor cells as a nutrient (16). Tyrosine is an important nurturing substance in malignant melanoma and glioblastoma multiforme (17, 18). Inhibition of tyrosine uptake could cut off the nutrient source to tumor cells, and when tumor cells are supplemented with tyrosine, tumor cells are able to regain their ability to proliferate (17). “Amino acid starvation therapy” has become a new approach for cancer treatment (19). Moreover, tyrosine catabolism significantly enhances the effects of chemotherapy, and the tyrosine metabolite fumaric acid, which has a unique role in suppressing translational DNA synthesis and improving chemosensitivity in ovarian cancer, can be used to improve the effects of chemotherapy through simple dietary supplementation with tyrosine (16). However, few studies have investigated the relationship between tyrosine and genes in lung cancer, and further analysis to explore the potential value of tyrosine metabolism in lung cancer is expected to provide new strategies for the prognostic prediction and treatment of LUAD. Therefore, exploring tyrosine-related genes (TRGs) has the potential to contribute to the construction of new sensitive and specific prognostic predictive biomarkers for LUAD patients.

In this study, we constructed prognostic prediction risk scores for TRGs on the basis of public biological databases and datasets, and a nomogram model was built to validate the sensitivity and specificity of these risk scores for prognostic prediction in LUAD patients. In addition, we investigated the risk scores in relation to tumor immunity, tumor mutation burden (TMB) and therapeutic sensitivity to antitumor drugs. The model was subsequently demonstrated to serve as a new very effective predictive biomarker of prognosis and treatment efficacy in LUAD patients.

## 2 Materials and methods

### 2.1 Data preparation

Gene expression and clinical information for LUAD patients, including a total of 59 normal and 541 LUAD patients, was retrieved from the TCGA database (https://portal.gdc.cancer.gov/). Valid clinical information was available for 507 patients. A total of 42 tyrosine metabolism genes were extracted and collected from the https://www.gsea-msigdb.org/gsea/msigdb/human/geneset/KEGG_TYROSINE_METABOLISM (**Supplementary Table 1**). The GSE68465 and GSE84437 https://www.ncbi.nlm.nih.gov/geo/datasets, which included 465 and 196 patients, respectively, were downloaded from GEO database.

### 2.2 Identification of differentially expressed genes

DEGs in the GSE68465 database were identified via the R package “limma”, and the screening criteria were adjusted *p* value< 0.05 and |logFC|>1.

### 2.3 Construction of the prognostic tyrosine-related risk score

The dataset was randomly divided into training and test groups at a 1:1 ratio. For Cox regression analysis, univariate and multivariate Cox regression analyses were performed sequentially to select the independent factors and avoid overfitting. Seven genes were ultimately identified for use in the construction of the prognostic risk model. The risk scores were calculated as follows:


$$risk\;scores=\sum_{i=1}^{n}mRNA_{i}\;Coef_{i}$$

Coefi and mRNAi symbolize the risk coefficient and gene expression, respectively. The patients were randomly divided into training and validation datasets at a 1:1 ratio on the basis of the risk scores. Patients in the training and validation cohorts were categorized into low-risk and high-risk groups, respectively, according to the median risk scores of the training cohort. K–M survival analysis was performed, and receiver operating characteristic (ROC) curves and the C-index were generated to explore the prognostic ability of the risk score.

### 2.4 Construction of the nomogram model

A nomogram was constructed via the R package “rms” on the basis of the matching score for each variable. Calibration plots were used to assess the predicted 1-, 3-, and 5-year OS rates against actual observations. Time-dependent ROC curves were plotted to assess the nomogram’s ability to predict 1-, 3-, and 5-year OS.

### 2.5 Functional analysis

Gene Ontology (GO) and Kyoto Encyclopedia of Genes and Genomes (KEGG) enrichment analyses were performed via the “limma”, “ggpubr” and “clusterProfiler” R packages. The filtering criteria were pvalue<0.05 and qvalue<0.05. Gene set enrichment analysis (GSEA) was used to explore potential functional and signaling pathway enrichment. The gene dataset “h.all.v7.1.symbols.gmt” was extracted from MSigDB.

### 2.6 Mutation and drug sensitivity analysis

Mutant gene frequencies were visualized via the R package “maftools”, and TMB was also calculated and plotted for the high- and low-risk score groups. Drug sensitivity analysis was performed via the R package “pRRophetic” to calculate the IC50 values of the antitumor drugs. Correlations were analyzed, and the relationships between the IC50 values and risk scores were identified.

### 2.7 Predictive analysis of immunotherapy response

Data concerning T-cell dysfunction, IFNG, CD8, MDSC, CAF, TAM M2, and TIDE scores were obtained from the Tumor Immune Dysfunction and Exclusion (TIDE) website (http://tide.dfci.harvard.edu/). The differences in the expression of these indicators were analyzed between the high- and low-risk groups and visualized through violin plots. TIDE was used to predict potential immune checkpoint blockade responses in LUAD. Higher TIDE scores are more likely to predict that the cells will evade immunization and decrease the immunotherapy response rate. Differences in immune cells and immune-related functions between the high- and low-risk groups were analyzed and presented as heatmaps via the “GSVA” package in R software.

### 2.8 MYO6 knockdown

The MYO6-siRNAs and the control-siRNAs were purchased from RiboBio (Guangzhou, China). The transfection process was guided according to the instructions of the jetPRIME@transfection reagent (Illkirch, France). The sequences of the MYO6 siRNAs used were as follows: siRNA-1, 5′-GAGGCUGCACU AGAUACUUUGCUAA-3′; and siRNA-2, 5′-GAGCCTTTGCCA TGGTACTTAGGTA-3′.

### 2.9 Cell scratch assay

A549 cells were inoculated into 6-well plates, after which MYO6 was knocked down. The cells were scraped with a pipette tip when they reached 100% confluence. Images were acquired at 0 or 24 h, and wound healing rates were analyzed via ImageJ software.

### 2.10 Apoptosis assay

The degree of cell apoptosis was determined with a Cell Apoptosis Analysis Kit (G3680, Solarbio, Beijing, China).

### 2.11 Statistical analysis

All the statistical analyses were performed via GraphPad 9.0 and R software. Differences between the two groups were calculated via two-sided t tests. Univariate and multivariate Cox regression analyses were used to explore the risk factors for prognosis in LUAD patients. Correlation analysis was performed via Spearman’s rank correlation. The Kaplan–Meier method was used to analyze and plot survival curves, and log-rank tests were performed to assess survival differences between the two groups. P< 0.05 was considered statistically significant.

## 3 Results

### 3.1 Clinicopathological characteristics

**Figure 1:**
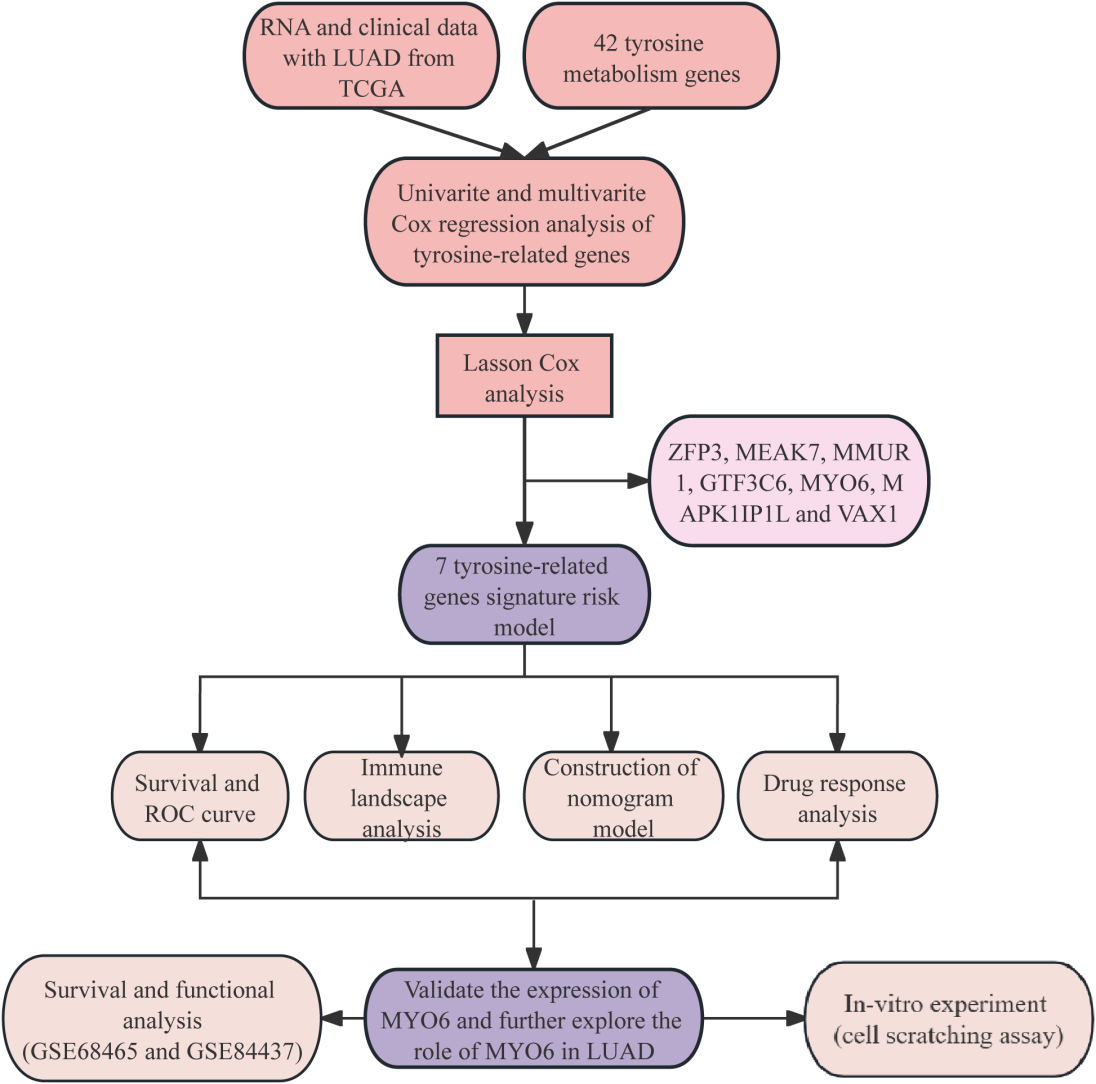
Flow chart of the research.

The analysis of the clinical information of 507 LUAD patients obtained from TCGA revealed that 239 (47.14%) patients were aged ≤65 years, and the proportion of female patients was 272 (53.65%). There were 272 (53.65%) LUAD patients diagnosed with stage I disease, 120 (23.67%) with stage II disease, 81 (15.98%) with stage III disease and 26 (5.13%) with stage IV disease. The highest percentages of patients were in the T2 and M0 stages, with 53.45% and 66.67%, respectively. There were 327 patients without lymph node metastasis (64.5%), 95 with lymph node metastasis (18.74%), 71 with N2 metastasis (14%), and 2 with N3 metastasis (0.39%). The clinical characteristics of these patients are summarized in **Supplementary Table 2**. The flow chart of the study is shown in **Figure 1**.

### 3.2 Screening of independent prognostic TRGs

**Figure 2:**
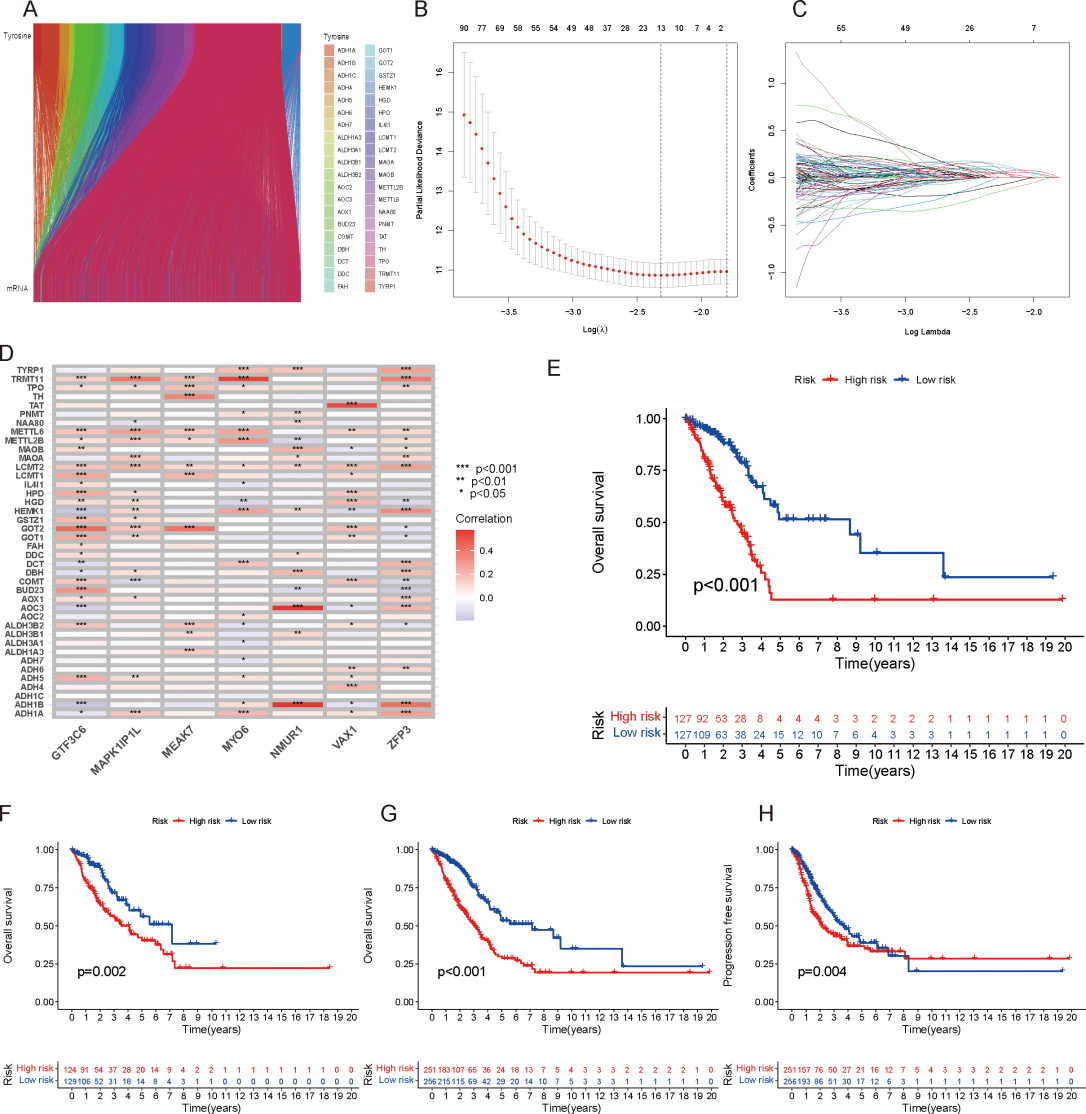
Screening of independent prognostic TRGs. **(A)** Sankey diagram showing the coexpression relationships between tyrosine-associated genes and TRGs. **(B)** Prognostic model establishment through LASSO Cox analysis. **(C)** Cross-validation of the minimum lambda value in the LASSO regression model. **(D)** Heatmap showing the correlations between these seven independent prognostic factors and TRGs, with red representing a positive correlation and green representing a negative correlation. **(E–G)**: OS survival analysis of different risk score groups in the training **(E)**, validation **(F)**, and total cohorts **(G)**. **(H)** PFS survival analysis of the total population.

In this study, 4429 TRGs were identified from 19,938 genes of LUAD patients and 42 tyrosine metabolism genes using |R|>0.1 and P<0.05 as the analytical criteria. The Sankey diagram shows the co-expression relationships between tyrosine metabolism genes and TRGs (**Figure 2**). LASSO Cox regression analysis was performed to prevent model overfitting (**Figures 2B**). In addition, univariate Cox regression studies identified 669 TRGs, and multivariate Cox analysis identified 7 TRGs, including ZFP3, MEAK7, MMUR1, GTF3C6, MYO6, MAPK1IP1L and VAX1, as independent prognostic factors (**Supplementary Table 3**). Risk scores were then calculated for each sample on the basis of the expression of the seven TRGs. Risk scores = (-0.333136591322958*ZFP3) + (0.611793040064573*MEAK7) + (-0.640417774274136*NMUR1) + (0.590932439411886*GTF3C6) + (-0.642310954703596*MYO6) + (0.903229361800426*MAPK1IP1L) + (0.460507883559416*VAX1). The heatmap shows the correlation between these seven independent prognostic factors and TRGs, with red representing a positive correlation and blue symbolizing a negative correlation (**Figure 2**). The patients were subsequently divided into high-risk and low-risk groups to better assess the prognostic value of the risk score. The results indicated that overall survival (OS) and progression-free survival (PFS) were significantly longer in the low-risk group than in the high-risk group in the training, test, and all groups (**Figures 2E**). These data suggest that the risk score has good predictive value and is a good prognostic signature.

### 3.3 Prognosis assessment of the risk score and establishment of a nomogram model

**Figure 3:**
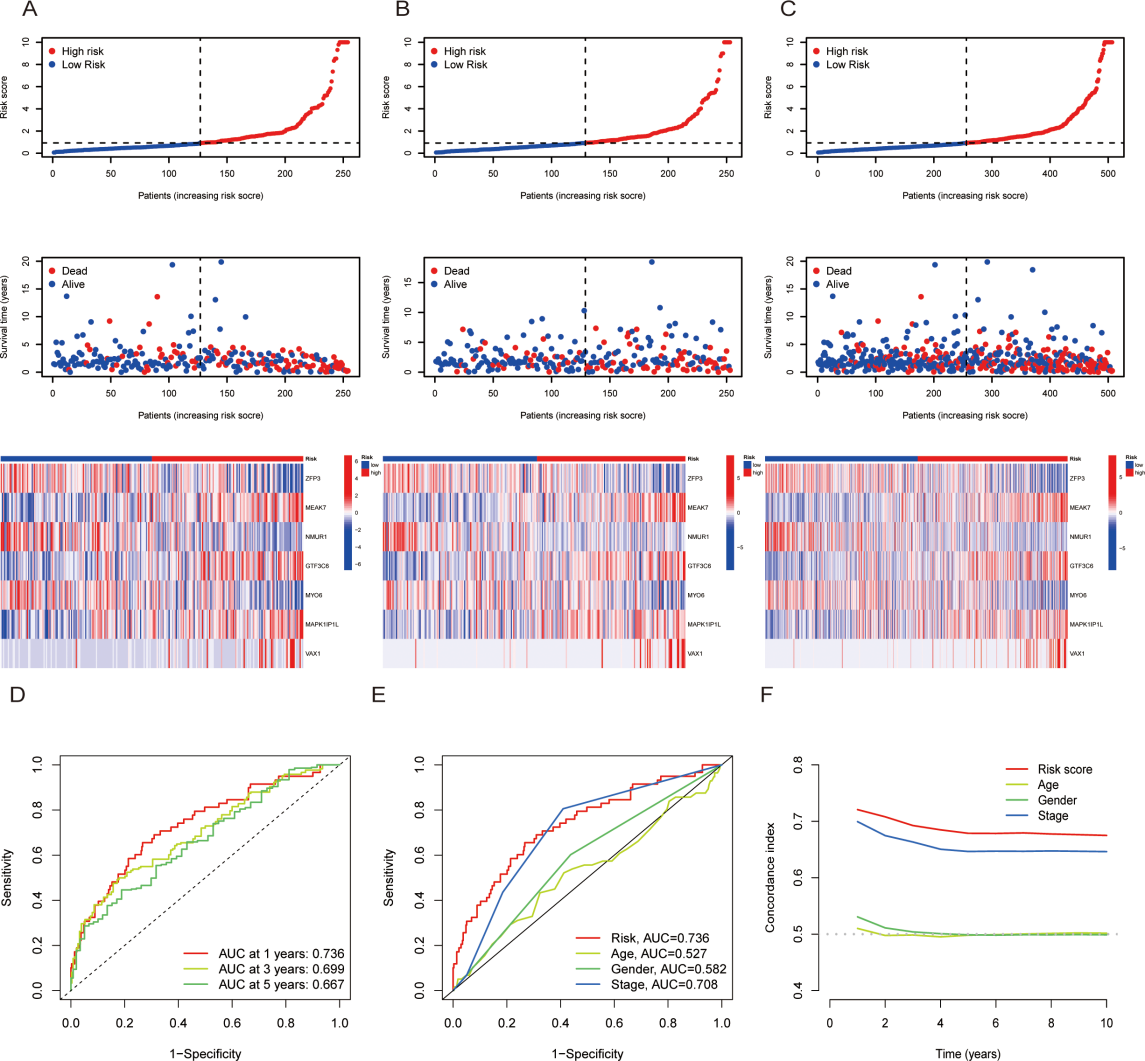
The prognosis assessment of the risk scores. **(A-C)**: Risk score curves, survival status plots and heatmaps between the high- and low-risk score groups in the training **(A)**, validation **(B)** and total cohorts. **(D)**: AUC curves of risk scores according to year. **(E)** AUC curves of the clinical indicators and risk scores. **(F)** Concordance index of clinical indicators and risk scores. AUC = area under the ROC curve.

To further validate the prognostic ability of the risk score, risk curves were plotted, which reflected the relationship between the risk score and survival status of LUAD patients. The results revealed that high-risk patients had higher mortality than low-risk patients did, and a heatmap revealed the distribution of seven TRGs between the high- and low-risk score groups (**Figures 3A**). In addition, we used receiver operating characteristic (ROC) curves to assess the predictive accuracy of the risk scores with respect to year and clinical characteristics. The AUC areas of 1-, 3- and 5-year OS were 0.736, 0.699, and 0.667, respectively; the AUC areas of risk scores and characteristics of age, sex and stage were 0.736, 0,527, 0.582, and 0.708, respectively, which indicated that the risk scores had high sensitivity and specificity (**Figures 3D**). Moreover, the C-index of the risk score was greater than that of other clinical characteristics, such as age, sex and stage (**Figure 3**). Additionally, OS was statistically significant for patients with high- and low-risk scores when classified by age, sex, and T stage, which demonstrated that patients in the low-risk score group had longer survival times (**Figures 1A**). Principal component analysis (PCA) was performed to observe the distribution of all the genes and TRGs among the LUAD patients, and the results revealed that the TRGs were clearly distributed (**Supplementary Figures 1G**). Therefore, the results above suggested that there was significant heterogeneity between high- and low-risk patients and that the risk score model had superior discriminatory power. The risk score has more reliable diagnostic value and can more accurately predict the prognosis of LUAD patients.

For the purpose of predicting the probability of patients’ outcome events or survival times more specifically. We included risk score, sex, age and stage for univariate and multivariate Cox regression analyses. The multivariate Cox regression results revealed that stage and risk score were independently associated with OS, suggesting that the signature of risk score was an independent prognostic factor for patients with LUAD (**Supplementary Figures 1I**). The nomogram model was subsequently constructed by integrating age, sex, stage and risk score (**Supplementary Figure 1**). The calibration curves revealed high predictive accuracy between the actual and simulated survival rates of patients at 1, 3, and 5 years (**Supplementary Figure 1**). Therefore, the nomogram model can be used to predict an individual and visualize the correlation between the total score and the predicted outcome more intuitively.

### 3.4 Potential mechanistic pathways associated with the risk score

**Figure 4:**
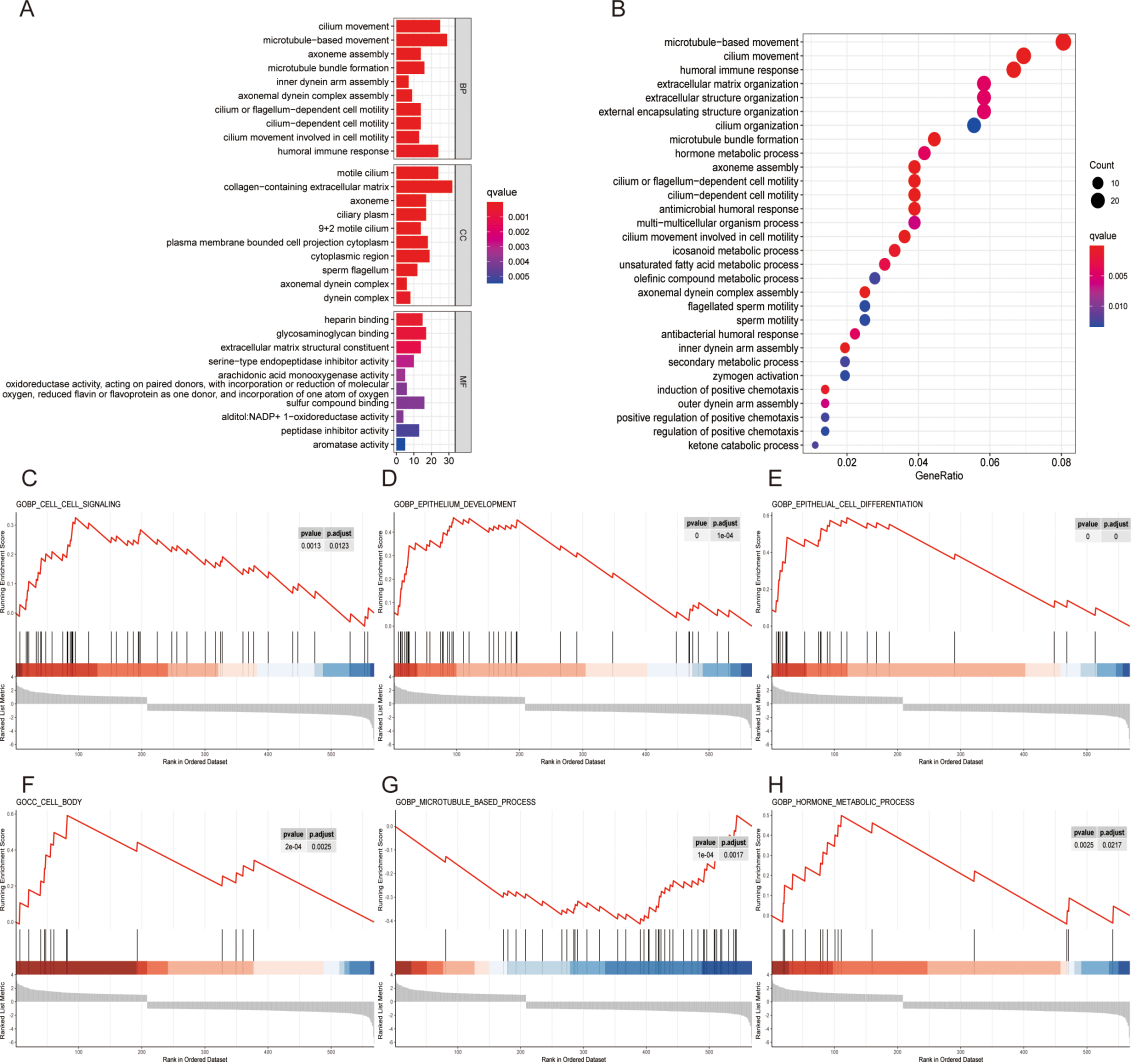
Exploration of potential mechanistic pathways. **(A)** GO enrichment analysis of the top ten mechanistic pathways for molecular function (MF), cellular component (CC) and biological process (BP). **(B)** KEGG enrichment analysis of the top 30 pathways in different risk groups. **(C-H)** Enrichment of functional pathways by GSEA in different risk score cohorts: cell signaling **(C)**, epithelium development **(D)**, epithelial cell differentiation **(E)**, cell body **(F)**, microtubule-based process **(G)** and hormone metabolic process **(H)**.

To explore the potential mechanistic pathways influenced by the risk score, GO functional enrichment analysis was performed, and the results revealed the top ten mechanistic pathways in terms of molecular function, cellular component and biological process (**Figure 4**). KEGG functional enrichment analysis indicated that the risk scores were associated with processes such as microtubule-based movement, cilium movement, the humoral immune response, extracellular matrix organization and extracellular structure organization (**Figure 4**). GSEA revealed that the risk score may be related to cell signaling, epithelial development, epithelial cell differentiation, the cell body, microtubule-based processes and hormone metabolic processes (**Figures 4C**). These results suggest that tyrosine metabolism may influence tumor progression by affecting cell development, differentiation and the immune system response.

### 3.5 Exploratory analysis: TMB, immune function and risk score

**Figure 5:**
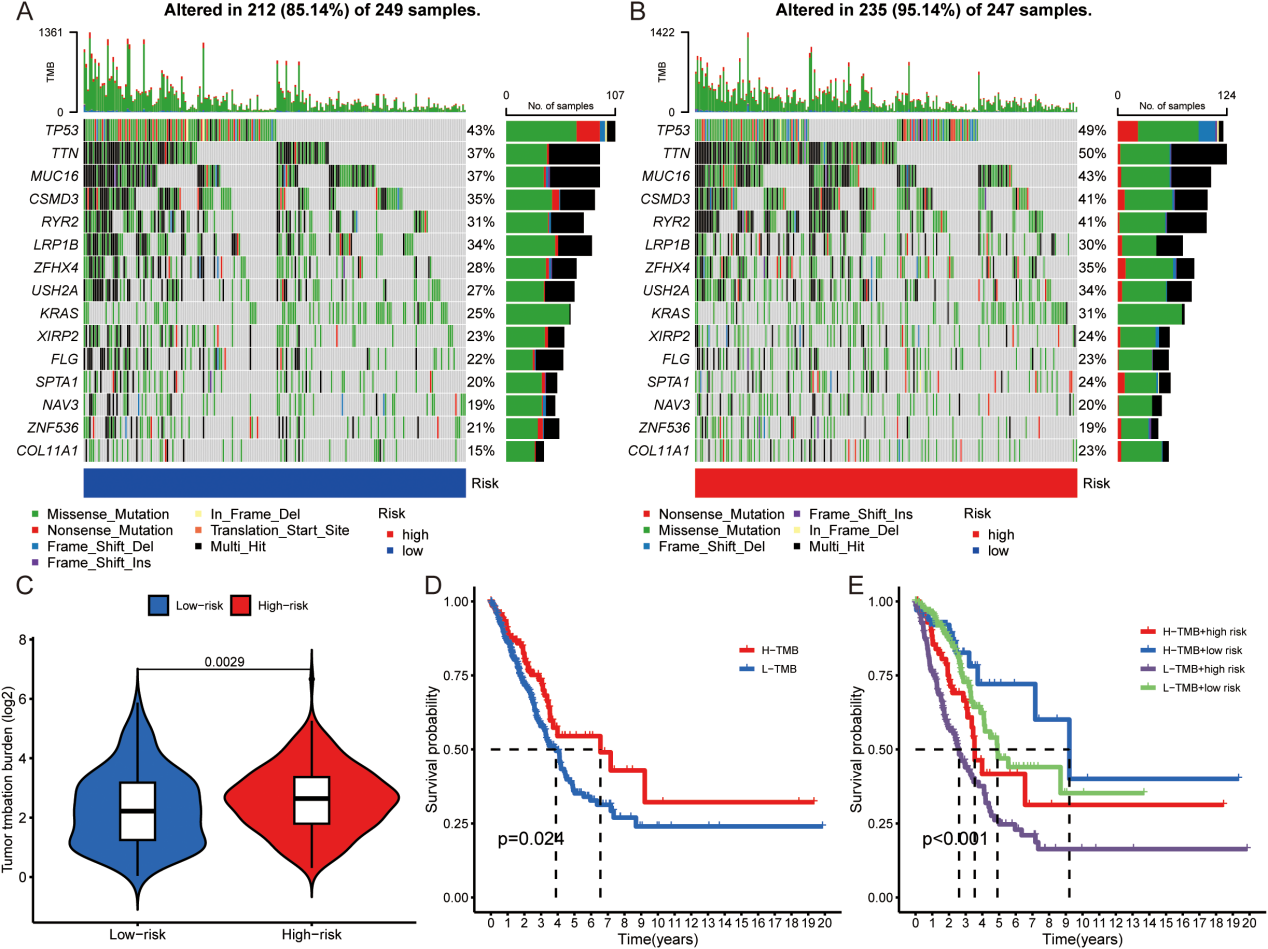
Comparison of TMB analysis. **(A, B)** Mutation landscapes of the low- **(A)** and high- **(B)** risk score groups. **(C)** TMB differences between high- and low-risk score patients. **(D)** Kaplan–Meier survival analysis according to TMB subtype. **(E)** Kaplan–Meier survival analysis of the TMB and risk score.

To explore analyze the specific pathways by which tyrosine affects the immune system, TMB analysis was carried out via the maftools algorithm in the high- and low-risk score groups. The maps revealed the mutation frequency distribution in the high- and low-risk score groups, and the results indicated that the number of mutation sites was greater in high-risk score patients than in low-risk score patients (such as TP53 high:49%: low 43%) (**Figures 5A**). The difference in TMB between the high- and low-risk score cohorts was statistically significant (**Figure 5**). We further investigated the difference in survival between patients with high and low TMB. OS was significantly better in the high-TMB group than in the low-TMB group (P = 0.024) (**Figure 5**). Compared with patients with low TMB and high-risk scores, patients with high TMB and low risk scores had better stratification when the two biomarkers were combined (**Figure 5**).

Furthermore, the relationships between immune-related functions, including immune cells and immune checkpoints, and risk scores were explored in depth. The heatmap revealed significant differences in immune function between high- and low-risk score patients in terms of the type II interferon (IFN) response, antigen-presenting cell (APC) costimulation, CCR, cytolytic activity, inflammation-promoting, T-cell costimulation, checkpoint, and T-cell coinhibition (**Supplementary Figure 2**). Differences in sensitivity to immunotherapy between patients in the high-risk and low-risk groups were further investigated via the TIDE (http://TIDE.dfci.harvard.edu/). The TIDE score is a tool developed for estimating the efficacy of tumor immune checkpoint therapies and is calculated on the basis of the presence of CAFs, MDSCs, and TAM M2 immune cells and the levels of CD8, IFNG, dysfunction and other indicators. The results demonstrated that the TIDE score was greater in the low-risk group than in the high-risk group (**Supplementary Figure 2**). When the expression of the individual variables that make up the TIDE score in patients with high- and low-risk scores was explored, the risk scores were significantly correlated with CAFs, MDSCs, TAM M2 immune cells, CD8+ T cells, IFNG+ cells and dysfunction (**Supplementary Figures 2C**). In summary, we conclude that the risk score may affect immune cell distribution, checkpoint expression and immunotherapy efficacy.

### 3.6 Exploratory analysis: drug response and sensitivity in different risk score groups

**Figure 6:**
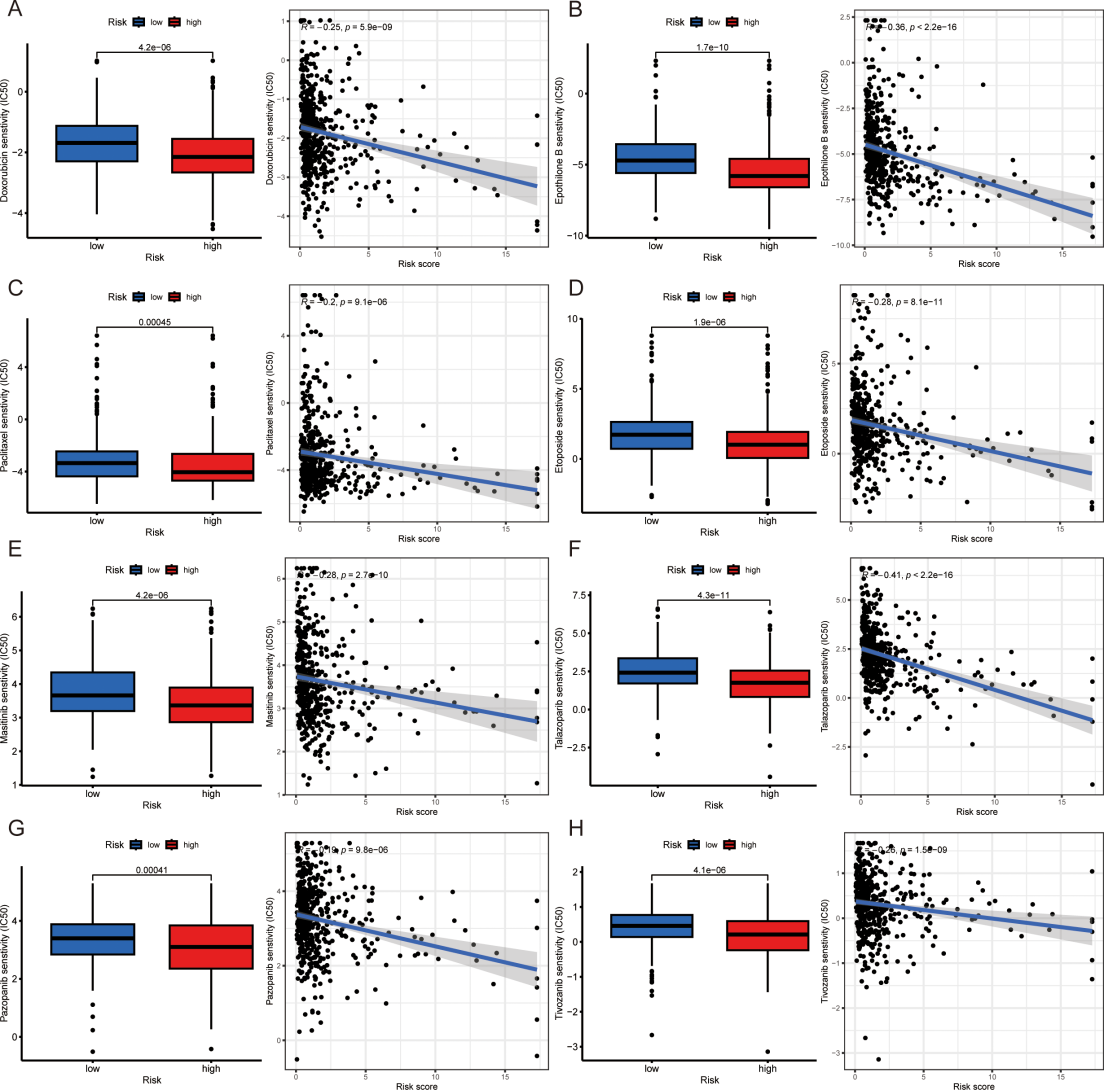
Drug sensitivity differences in patients with various risk scores. **(A-H)** Drug sensitivity disparities and correlations between high- and low-risk score groups: doxorubicin **(A)**, epothilone B **(B)**, paclitaxel **(C)**, etoposide **(D)**, masitinib **(E)**, talazoparib **(F)**, pazopanib **(G)** and tivozanib **(H)**. *IC50: half maximal inhibitory concentration.

To predict the clinical therapeutic response to drugs in patients with different risk scores, we used the “pRRophtic” package to screen for potentially effective antitumor drugs. There was a close correlation between the risk score and drug sensitivity to doxorubicin, epothilone B, paclitaxel, etoposide, masitinib, talazoparib, pazopanib and tivozanib. Patients with higher risk scores had lower IC50 values (concentrations that inhibited cell growth by 50%), suggesting that patients with high-risk scores are more sensitive to these drugs (**Figure 6A**). It is reasonable to speculate that antitumor effects can be acquired by low doses of these drugs.

### 3.7 MYO6 inhibits tumor proliferation in NSCLC

**Figure 7:**
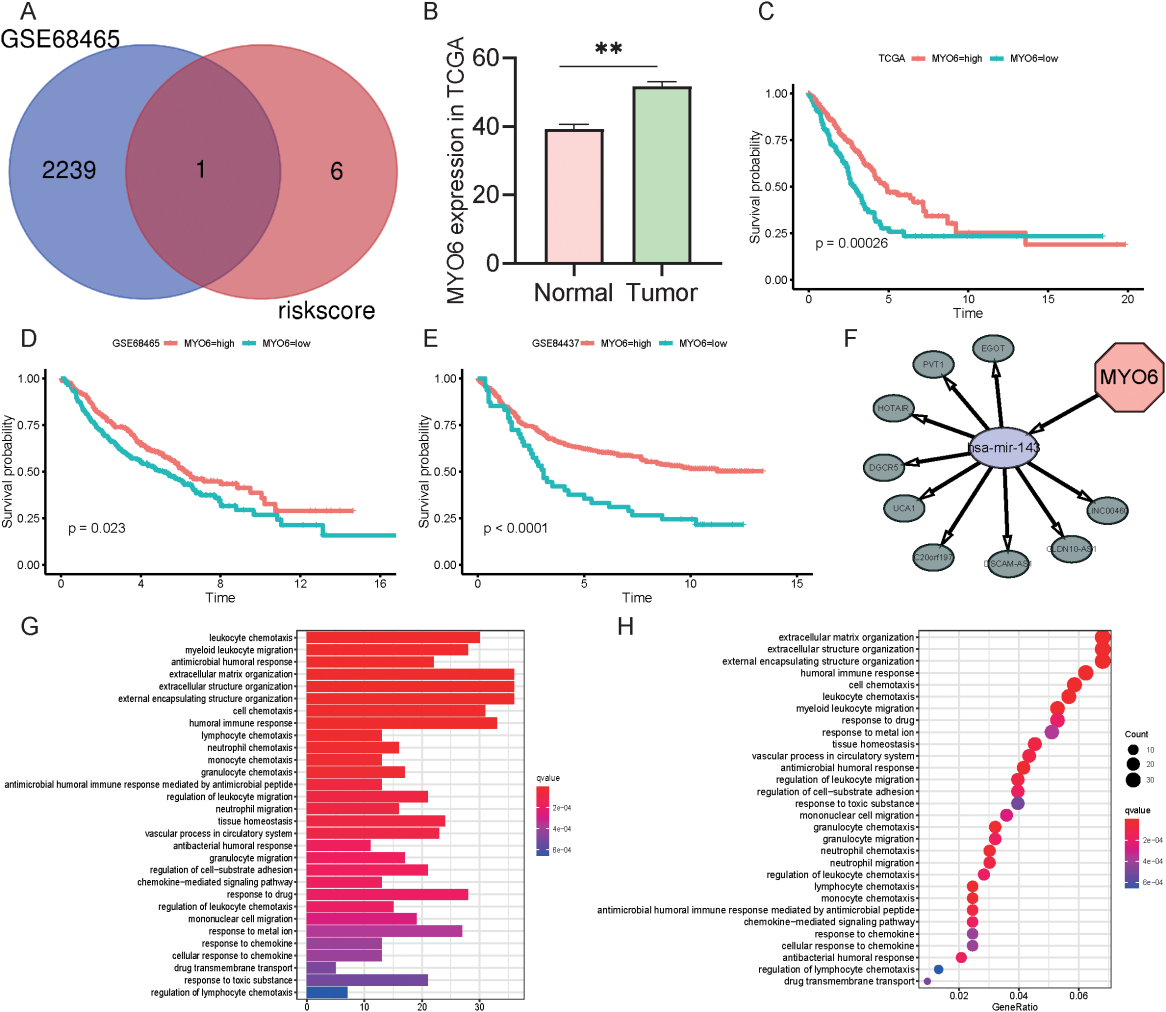
Role and function of MYO6 in NSCLC. **(A)** Venn diagram of the DEGs in GSE68465 and seven TRGs. **(B)** The expression of MYO6 in the TCGA cohort (normal group n=59, tumor group n=541). **(C-E)** Kaplan-Meier analysis compared the survival of high- and low-risk groups (defined by the optimal cut-off) in the TCGA (n=365 high vs. n=142 low) **(C)**, GSE68465 (n=217 vs. n=225) **(D)**, and GSE84437 (n=386 vs. n=47) **(E)** cohorts. **(F)** Construction of ceRNA networks using MYO6 as a target gene. **(G)** GO enrichment analysis using MYO6 as a target gene. **(H)** KEGG enrichment analysis using MYO6 as a target gene.

The above results demonstrated that the genes comprising the risk scores influenced the development of LUAD to some extent. To better explore the effects of specific genes on LUAD, we downloaded the GSE68465 dataset from the GEO database. The GSE68465 dataset is a multisite, blinded validation study describing gene expression-based survival prediction in LUAD. A Venn diagram revealed the intersection between the DEGs in GSE68465 and the independent factors of seven TRGs; MYO6 was the only gene present in both datasets (**Figure 7**). To explore the potential antitumor mechanism of MYO6, we found that MYO6 was highly expressed in tumors in the TCGA cohort (**Figure 7**). Patients with high expression of MYO6 in the TCGA cohort had a poor prognosis, which was validated in the GSE68465 and GSE84437 datasets (**Figure 7C**). In addition, TCGA data were utilized to construct ceRNA networks using MYO6 as a target gene. MYO6 can target hsa-mir-143 and affect the expression of related lncRNAs, such as EGOT and PVT1 (**Figure 7**). The potential mechanism of MYO6 explored via GO and KEGG analyses revealed that the five pathways with the greatest influence were extracellular matrix organization, extracellular structure organization, external encapsulating structure organization, the humoral immune response and cell chemotaxis (**Figure 7G**).

**Figure 8:**
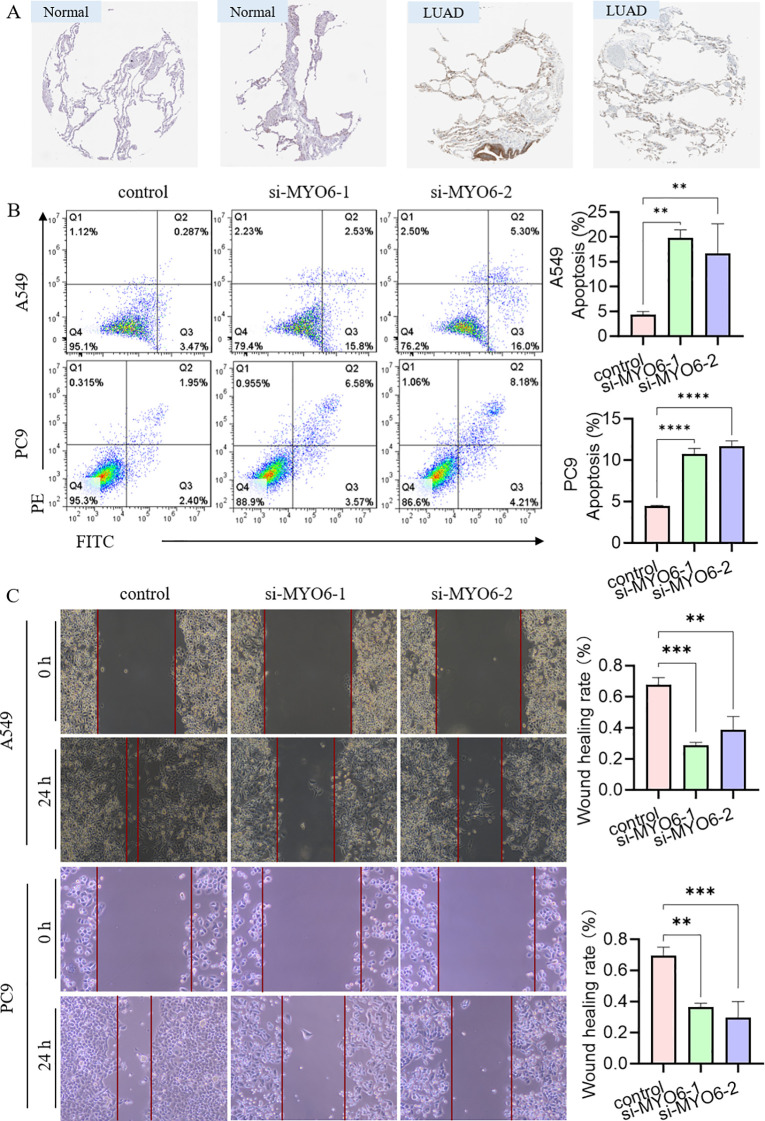
The effect of MYO6 on tumor proliferation in NSCLC. **(A)** Images showing the protein expression of MYO6 in normal tissue and LUAD. **(B)** The cell proliferation ability of A549 and PC9 cells was detected via an apoptosis assay after MYO6 knockdown (mean ± SD, n = 3). **(C)** The migration rates of A549 and PC9 cells were evaluated via a scratch assay after MYO6 knockdown (mean ± SD, n = 3).

We also analyzed the protein expression of MYO6 in clinical LUAD samples and normal samples via the Human Protein Atlas (HPA) database (www.proteinatlas.org) and reported that the protein expression level of MYO6 was significantly greater in tumor tissue than in normal lung tissue (**Figure 8**). The efficacy of MYO6 knockdown by the specific siRNA was confirmed using quantitative PCR (qPCR) and Western blot (WB) (**Supplementary Figure 3A**). The function of MYO6 in cancer was subsequently explored via *in vitro* experiments. The results of the apoptosis assay revealed that the knockdown of MYO6 could influence cell proliferation in LUAD (**Figure 8**). Cell scratch assays demonstrated that MYO6 knockdown significantly reduced the migration (**Figure 8**). These results suggest that MYO6 promotes tumor proliferation and migration and plays an oncogenic role in lung cancer.

## 4 Discussion

Amino acids are among the biologically active macromolecules that build biological organisms and play important roles in malignant tumors (20, 21). Disorders of amino acid metabolism can lead to the deficiency or accumulation of fats, enzymes and vitamins and the disruption of homeostatic mechanisms, which have profound effects on malignant cell dynamics and the tumor immune response (22). Tyrosine, as one of the 20 amino acids that synthesize proteins in the body, is used mainly for nutritional support, metabolism, growth and development and can be used as a biomarker for early screening and risk assessment of malignant tumors (23, 24). Studies have shown that most patients with malignant tumors have significantly increased concentrations of amino acids in the urine (up to 50–150%) (25). Therefore, the metabolic activity of human malignant tumor cells could be evaluated by detecting tyrosine levels in human urine (26). EGFR is a tyrosine kinase receptor, the EGFR inhibitor CL-387785 suppressed the invasion and metastasis of H1975 cells (27). Currently, oncogene-based prognostic risk signatures lack ideal predictive ability, and a clear method for connecting tyrosine with tumor genes to establish a prognostic model for lung cancer is lacking. Therefore, it is important to construct more reliable prediction models to assess the prognosis and survival of LUAD patients.

In this study, we constructed a TRG prognostic model based on the co-expression network of mRNAs in TCGA and tyrosine metabolism genes, thus exploring the underlying mechanisms. Seven independent prognostic TRGs were subsequently identified in patients with lung cancer via univariate and multivariate regression analyses. The risk score was established by the 7 TRGs above, and patients were classified into high- and low-risk groups according to the risk scores. Moreover, the prognosis of LUAD patients in the high- and low-risk score groups could be stratified more accurately through survival analysis, distribution of patient survival status, receiver operating characteristic (ROC) curves, and principal component analysis (PCA), and patients in the high-risk score group were closely associated with shorter survival times. These results demonstrated that the risk score established by seven TRGs is a highly sensitive and specific biomarker for predicting survival time and prognostic outcomes in patients with LUAD.

Among these seven independent prognostic TRGs, MYO6, the only reverse-direction motor protein moving toward the minus end of actin filaments, is involved in the genesis and progression of multiple tumors (28). MYO6 is highly expressed in prostate cancer, ovarian cancer, gastric cancer and oral squamous cell carcinoma and can promote proliferation and migration through the lncRNA UCA1/miR-143/MYO6 axis (29–32). MYO6 knockdown inhibited cell proliferation, migration, and invasion and induced apoptosis in colon cancer through Circ_0011385/miR-330-3p/MYO6, the lncRNA SOX21-AS1/miR-145/MYO6, HNF1A-AS1/miR-124/MYO6, and the CircCSNK1G1/miR-455-3p/MYO6 axis (33–36). MYO6 knockdown is associated with disease progression, poor prognosis and immune cell infiltration in clear cell renal cell carcinoma (37). Knocking down MYO6 could influence cell growth and proliferation in lung cancer through the phosphorylation of ERK1/2 and the microRNA-5195-3p/MYO6 axis (38, 39). These studies provide a theoretical basis for the involvement of MYO6 in the pathogenesis of colorectal cancer and suggest that MYO6 may be a useful biomarker for cancer treatment. The results of this study are consistent with previous conclusions that MYO6 expression is upregulated in NSCLC tumors and MYO6 promotes tumor migration in lung cancer. The ceRNA network suggested that MYO6 may target miR-143 and thus affect the expression of EGOT or PVT1, etc. The potential mechanism of its influence on lung cancer may involve extracellular matrix and structural organization, external encapsulating structure organization, the humoral immune response and cellular chemotaxis. Moreover, our results showed that MYO6 could affect the apoptotic process and metastasis *in vitro*. However, its specific regulatory mechanism needs to be further studied. Interestingly, the apparent contradiction between its negative coefficient in the multivariate risk model and its pro-oncogenic experimental validation reflects the complexity of cancer biology. The reasons may be blow: 1) the negative coefficient may result from its statistical interactions with other covariates in the multivariate model rather than its intrinsic biological function; 2) potential discordance between mRNA expression and protein activity may arise from post-transcriptional/translational regulation; 3) context-dependent roles of MYO6 in different cellular environments could contribute to this discrepancy.

ZFP3 is localized to chromosomes 17p12–17pter46, and research reports have demonstrated the prognostic role of ZFP3 in head and neck squamous and lung cancers (40–42); however, exploration of its underlying mechanisms is lacking. mEAK-7 is significantly elevated in the tumors and metastatic lymph nodes of NSCLC patients, and microRNA-1911-3p can target mEAK-7 to inhibit mTOR signaling in human lung cancer cells (43, 44). However, the role of mEAK-7 in human cancers has not been determined, and the downstream regulation of mEAK-7 in human cells is still unclear. NMUR1 is a neuropeptide associated with energy homeostasis and tumor progression, and renal cancer cells can express functional NMUR1 and stimulate tumor migration (45). High NMUR1 expression was associated with shorter OS in CRC patients (46). Inhibition of NMUR1 upregulated the antitumor activity of CD8^+^ T cells and the glycolytic process of tumor cells in the tumor microenvironment of pancreatic ductal adenocarcinoma (47). Bioinformatic approaches revealed NMUR1 as a novel key pathogenic gene in luminal A breast cancer, which may be a potential target for clinical therapy (48). However, its role and potential mechanisms in lung cancer remain unexplored. There are fewer studies on GTF3C6, MAPK1P1K and VAX1. Researchers have indicated that a 15-characteristic gene, including GTF3C6, may affect the prognosis of hepatocellular carcinoma (49). A study of five urinary biomarkers, including MAPK1IP1L, may serve as a basis for the adjunctive diagnosis of lung cancer (50). VAX1 is associated with bladder cancer recurrence, and a combination of 8 genes, including VAX1, can be used as a diagnostic marker for monitoring bladder cancer disease progression (51). The functions of these TRGs contribute to the understanding of the pathogenesis and progression mechanisms of LUAD and provide new potential targets for the treatment of LUAD.

In addition, the relationships among immunity, TMB, and risk score were analyzed among patients with LUAD, and the results revealed that the II-IFN response, HLA-APC costimulation, inflammation-promoting, T-cell costimulation, T-cell coinhibitory, and checkpoint effects were significantly greater in the high-risk group than in the high-risk group. II-IFN is a cytokine with both antitumor and protumor activity that can be used as a nexus for immunotherapy response (52). Immunotherapy-induced IFN produces conflicting antitumor or protumor effects, and its expression is rationally utilized to bias its antitumor effects and to avoid immune escape (53). HLA is an expression product of the human histocompatibility complex, which participates in the regulation of a variety of immune cells, including macrophages, and is also a key factor in the immunotherapy response (54). APC, a tumor suppressor gene, mainly regulates cell proliferation and migration (55). These immune features partly explain the poorer prognosis of patients in the high-risk group. It is reasonable to hypothesize that the risk score may have an impact on immune cell distribution, checkpoint expression and immunotherapy efficacy. Moreover, our results revealed that the mutation frequency was greater in the high-risk group than in the low-risk group, and when we combined TMB and risk scores, the patients could be stratified better, indicating good prognostic value. TMB, a detection method used to assess the number of somatic nonsynonymous mutations in a specific region, is a hotspot marker for evaluating the therapeutic effect of immunochemotherapy (56). TP53, a component of the TMB calculation, has a high mutation rate in lung cancer (57). TP53 mutation can play a direct role in promoting cancer development, and TP53 mutation may be correlated with chemoresistance and immune resistance (58).

In this study, we characterized the TRG signatures in LUAD and established a risk-prognostic model consisting of seven TRGs, which provides a new theoretical basis for the diagnosis and treatment of LUAD patients. Our analysis demonstrates distinctness from existing models, previously reported immune or metabolic signatures share no gene overlap with our 7 TRGs (9, 59–63). While other metabolic studies exist (59, 61), none specifically focus on tyrosine metabolism. Thus, our work presents the LUAD prognostic signature from this unique perspective, with its distinct biology and different gene composition strongly underscoring its novelty. However, there were several limitations to this study. First, clinical information, such as information related to immunotherapy and targeted therapy, was missing in this study and could not be analyzed for the effects of tyrosine metabolism-related genes on the prognosis of patients receiving immunotherapy. Secondly, we lack tissues from patients with clinical LUAD to detect tyrosine metabolism-related protein expression. Therefore, the clinical implications of our findings require further validation. Prospective analyses incorporating clinical samples with complete immunotherapy records are essential to confirm the prognostic value of our signature. Functional validation in patient-derived xenograft models or other *in vivo* systems will be critical to bridge our bioinformatic discoveries to potential clinical applications.

## Conclusion

In summary, the risk scores constructed from the seven TRGs have great potential for survival prognosis, immunotherapy response and drug sensitivity. MYO6 plays an oncogenic role in promoting proliferation and metastasis in patients with LUAD, which provides a new theoretical basis for the diagnosis and treatment of LUAD patients.

## Data Availability

The original contributions presented in the study are included in the article/**Supplementary Material**, further inquiries can be directed to the corresponding author/s.

## Supplementary material

Supplementary Table 1 Tyrosine-related genes.

Supplementary Table 2 Clinicopathological characteristics of LUAD patients in the TCGA dataset.

Supplementary Table 3 Seven tyrosine-related mRNAs identified by multivariate Cox analysis.

Supplementary Figure 1 Construction of the nomogram model. **(A-F)** Kaplan–Meier survival analysis of different risk score groups according to age ≤ 66 years **(A)**, age > 66 years **(B)**, female sex **(C)**, male sex **(D)**, T1 stage **(E)**, and T2–4 stage **(F)**. **(G)** PCA of all genes; **(H)** PCA of TRGs; **(I)** univariate Cox regression analysis. **(J)** Multivariable Cox regression analysis. **(K)** Construction of the nomogram. **(L)** 1-, 3-, and 5-year calibration graphs for the evaluation of nomogram accuracy.

Supplementary Figure 2 Immune-related functional analysis. **(A)** Heatmap of immune function between high- and low-risk score patients. **(B)** Differences in TIDE expression across risk score groups. **(C-H)** Differences in the distributions of immune-related signatures, including CAFs **(C)**, MDSCs **(D)**, M2 TAMs **(E)**, CD8+ T cells **(F)**, IFNG+ cells **(G)**, and dysfunction **(H)**, between high- and low-risk patients.

Supplementary Figure 3 qRT-PCR and Western Blot were employed to detect siRNA knockdown efficiency in A549 cell line (mean ± SD, n = 3).
