# Supplementary figures and images for: Application of a risk score model based on tyrosine-related genes in the prognosis and treatment of patients with lung adenocarcinoma
Source: Front Immunol. 2025 Nov 4;16:1667473. doi: 10.3389/fimmu.2025.1667473 (PMC12623329; doi:10.3389/fimmu.2025.1667473)

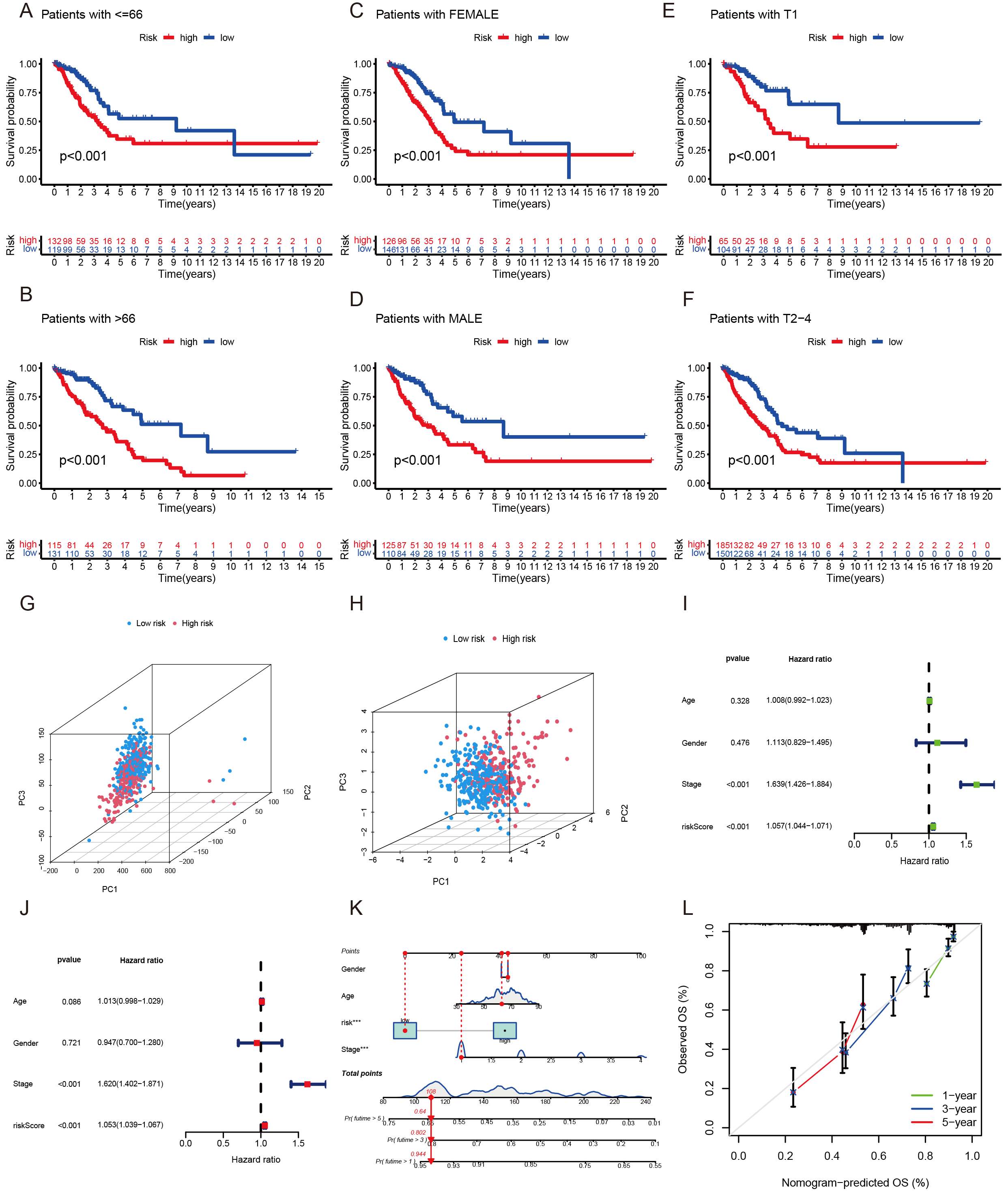

Supplement: Supplementary Figure 1 — Construction of the nomogram model. (A-F) Kaplan–Meier survival analysis of different risk score groups according to age ≤ 66 years (A), age > 66 years (B), female sex (C), male sex (D), T1 stage (E), and T2–4 stage (F). (G) PCA of all genes; (H) PCA of TRGs; (I) univariate Cox regression analysis. (J) Multivariable Cox regression analysis. (K) Construction of the nomogram. (L) 1-, 3-, and 5-year calibration graphs for the evaluation of nomogram accuracy. [file Image1.tif]

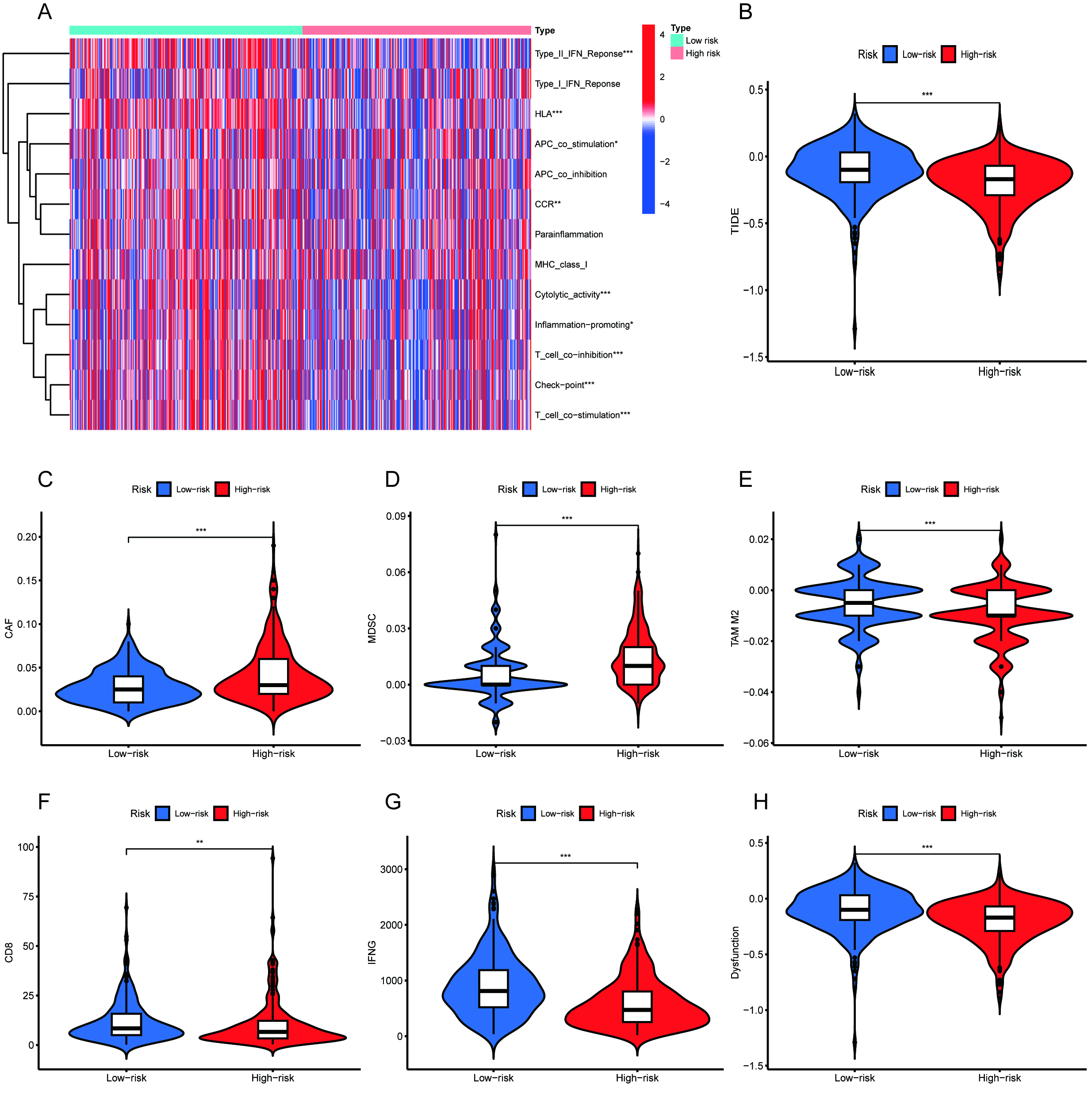

Supplement: Supplementary Figure 2 — Immune-related functional analysis. (A) Heatmap of immune function between high- and low-risk score patients. (B) Differences in TIDE expression across risk score groups. (C-H) Differences in the distributions of immune-related signatures, including CAFs (C), MDSCs (D), M2 TAMs (E), CD8+ T cells (F), IFNG+ cells (G), and dysfunction (H), between high- and low-risk patients. [file Image2.tif]

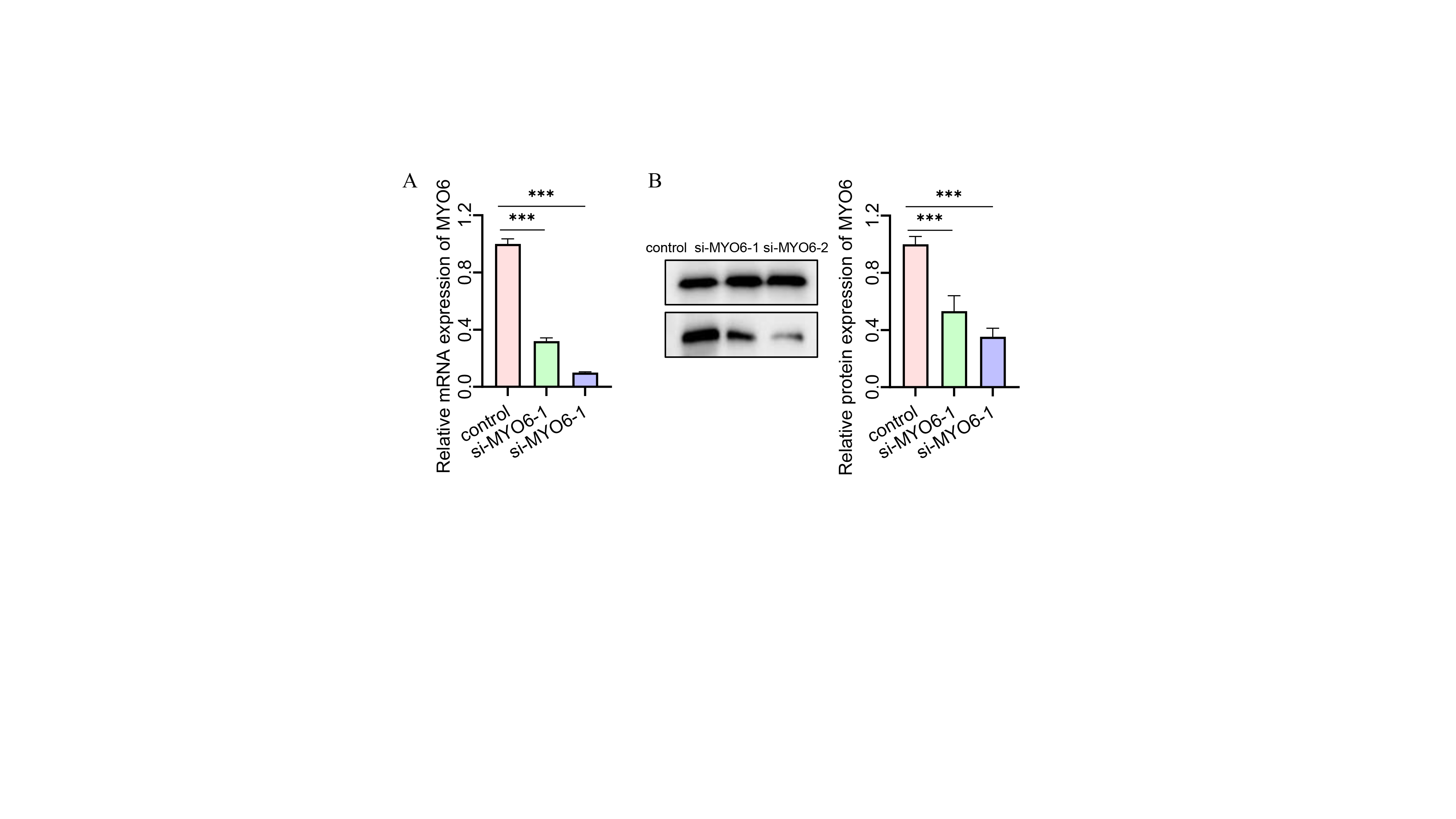

Supplement: Supplementary Figure 3 — qRT-PCR and Western Blot were employed to detect siRNA knockdown efficiency in A549 cell line (mean ± SD, n = 3). [file Image3.tif]
